# Beyond Static Representations: Learning Electroencephalogram Spatiotemporal Dynamics for Enhanced Emotion Recognition

**DOI:** 10.34133/cbsystems.0701

**Published:** 2026-10-28

**Authors:** Ke Liu, Wenrui Zhu, Rui Xu, Wenlong Wang, Zhuliang Yu, Wei Wu

**Affiliations:** ^1^ Chongqing Key Laboratory of Computational Intelligence, Chongqing University of Posts and Telecommunications, Chongqing 400065, China.; ^2^ Key Laboratory of Big Data Intelligent Computing, Chongqing University of Posts and Telecommunications, Chongqing 400065, China.; ^3^ School of Computer Science and Technology, Chongqing University of Posts and Telecommunications, Chongqing 400065, China.; ^4^ Department of Neurology, Songjiang Hospital and Songjiang Research Institute, Shanghai Key Laboratory of Emotions and Affective Disorders, Shanghai Jiao Tong University School of Medicine, Shanghai 201600, China.; ^5^ College of Automation Science and Engineering, South China University of Technology, Guangzhou 510641, China.

## Abstract

Electroencephalogram (EEG)-based emotion recognition plays a pivotal role in brain–computer interfaces and mental health monitoring. However, conventional approaches often neglect the intrinsic spatial correlations among EEG channels and fail to effectively model both global temporal dependencies and the continuity of neural oscillations. To overcome these challenges, we propose the Dynamic Spatial Embedding and Temporal Attention Mamba (DSE-TAM) framework. Our Dynamic Spatial Embedding (DSE) module leverages graph convolutional networks to dynamically capture inter-channel relationships, preserving spatial coherence in EEG representations. Subsequently, the Temporal Attention Mamba module integrates self-attention with state-space models to jointly optimize long-range temporal dependencies and smooth transition dynamics. Extensive experiments on 2 public benchmarks (DEAP and DREAMER) and an in-house CEED dataset demonstrate that DSE-TAM surpasses state-of-the-art methods, achieving accuracies of 79.75% (arousal) and 78.27% (valence) on DEAP, 80.55% (arousal) and 83.91% (valence) on DREAMER, and 73.75% (3-class) on CEED. These results validate the framework’s efficacy in advancing EEG-based emotion decoding for brain–computer interfaces. The source code is available at https://github.com/Raizeroko/DSE-TAM.

## Introduction

Emotion substantially impacts cognition, behavior, and communication, making its analysis and recognition a vital research area. Historically, external input, such as facial expressions, speech, and posture, is easy to obtain, but reliability diminishes when individuals intentionally hide their emotions or experience emotional disorders. An electroencephalogram (EEG), due to its noninvasive nature and high temporal resolution arising from cortical electrical activity, offers a reliable data source for objective emotion recognition. Consequently, EEG-based emotion analysis is a prominent research area in affective brain–computer interfaces [1].

Machine learning has emerged as a powerful tool for tackling problems that involve large amounts of data, providing flexible and scalable solutions. Recent studies continue to optimize traditional and hybrid machine learning pipelines to establish robust benchmarks. For example, comprehensive framework studies like the TorchEEGEMO toolbox [2] and the LibEER platform [3] highlight that optimized feature extraction combined with classical classifiers remains highly competitive and offers scalable solutions alongside deep learning architectures. Furthermore, advanced traditional models, such as sparse Bayesian learning frameworks, have recently shown strong capabilities in handling feature space variations across subjects [4]. In addition, Cheng et al. [5] introduced the gcForest deep forest model, known for fewer hyperparameters and adaptability to different dataset sizes, offering robust performance for EEG-based emotion recognition.

Deep neural networks have been applied to information extraction from handcrafted features, such as power spectral density and differential entropy (DE) [6–8]. In addition, prior studies have systematically analyzed the importance of features, frequency bands, and channels in EEG-based emotion recognition, further demonstrating the critical role of informative EEG representations [9]. Although these approaches have shown promise, their reliance on manually engineered feature maps as input may hinder full exploitation of the end-to-end representation learning capabilities of deep learning. Furthermore, handcrafted features can inadvertently discard critical information embedded within the raw EEG signals [10]. Therefore, methods capable of directly modeling raw EEG data are needed to effectively preserve the inherent spatiotemporal dynamics and structural information contained within the signals.

To facilitate feature extraction from raw EEG data, embedding techniques are employed to transform the high-dimensional signals into compact, low-dimensional feature spaces. This dimensionality reduction process aids in discerning salient temporal, spectral, and spatial information inherent within the EEG recordings [11,12]. A prevalent strategy involves leveraging convolutional operations to generate token-like representations from EEG signals, analogous to embedding methods in natural language processing. These tokens effectively encapsulate key spatial features present in the EEG data, providing a structured input amenable to sophisticated temporal modeling for emotion recognition tasks [13,14]. However, many current models segment raw EEG data into fixed-length blocks on a per-channel basis. This channel-wise segmentation, which treats each channel as an independent sequence [12,15], inherently disrupts the intrinsic spatial correlations and global topological structure existing among the channels. Consequently, the model’s capacity to comprehensively capture the complex spatiotemporal dynamics of EEG signals may be constrained.

Temporal representation learning plays a pivotal role in EEG-based emotion decoding, leveraging tokenized embeddings as input. Recurrent neural networks have been widely employed to model sequential dependencies, owing to their inherent temporal inductive bias [16]. However, recurrent neural networks often encounter limitations in capturing long-range temporal dependencies due to the vanishing gradient problem [17,18]. Self-attention mechanisms [19,20] offer an alternative by enabling global receptive fields, yet their permutation-invariant nature can compromise temporal coherence. This is particularly relevant for high-temporal-resolution EEG data, where preserving the continuity of neural oscillations associated with emotional processes is crucial [21]. Consequently, effective emotion decoding necessitates methods that can both maintain spatial embedding integrity and robustly model temporal dynamics.

To address the aforementioned limitations and enable a comprehensive representation of EEG spatiotemporal dynamics, we propose the Dynamic Spatial Embedding and Temporal Attention Mamba (DSE-TAM) model. The key idea of the Dynamic Spatial Embedding (DSE) module is to embed graph-based spatial learning into the early feature extraction pipeline of raw EEG signals. Instead of treating EEG channels as independent temporal sequences or generating tokens solely through conventional convolutional operations, DSE introduces a learnable adjacency matrix to adaptively infer inter-channel dependencies during the embedding process. The graph-filtered multichannel EEG segments are then transformed into a temporally ordered sequence of compact EEG tokens. In this way, the spatial topology of brain activity is incorporated before temporal modeling, allowing each token to encode both local temporal information and graph-guided spatial interactions. This design preserves the temporal continuity of EEG signals while enhancing the spatial discriminability of the learned representations, thereby establishing a robust foundation for subsequent temporal modeling.

Complementing the spatial embeddings generated by DSE, our temporal feature extractor comprises a Shallow Temporal Mamba (STM) module followed by a stacked Temporal Attention Mamba (TAM) module. Recent advancements in EEG analysis have demonstrated the efficacy of Mamba-based architectures in capturing the long-range dependencies and nonstationary dynamics characteristic of neural time-series data [22,23]. In this work, the STM module operates on the spatially embedded token sequence to extract fine-grained temporal features at a high resolution. The TAM module further enhances temporal modeling by integrating self-attention mechanisms with state-space models (SSMs).

This hybrid architecture ensures robust modeling of both short-term fluctuations and long-range temporal interactions while maintaining the physiological continuity of EEG signals. Extensive empirical evaluations on 2 widely used public benchmarks (DEAP and DREAMER) as well as a proprietary EEG dataset demonstrate the superior performance of the proposed DSE-TAM framework.

In summary, the main contributions of this work are 2-fold:

• We propose a DSE module that integrates graph convolutional networks (GCNs) into the EEG representation learning pipeline. Unlike conventional convolutional neural network (CNN)-based embedding methods that rely on convolutional operations to capture spatial features during the embedding process, our approach employs a learnable adjacency matrix to adaptively infer the intrinsic spatial dependencies among EEG channels, achieving EEG embedding while preserving the temporal coherence of the sequences. This innovation enables a unified spatiotemporal representation while substantially enhancing the discriminative capacity of spatial features for emotion recognition.
• To address the challenges of modeling EEG’s nonstationary and long-range temporal dynamics, we introduce a TAM module that combines SSMs with self-attention mechanisms. This hybrid design captures both global contextual dependencies (via attention) and smooth dynamic transitions (via SSMs), effectively balancing computational efficiency with physiological plausibility.

The remainder of this paper is organized as follows: The proposed method is described in detail in Methods. Experiments elaborates on the experimental protocol, and Results presents the results. The discussion is provided in Discussion, followed by a brief conclusion in Conclusion.

## Methods

The proposed DSE-TAM model, illustrated in Fig. 1, consists of 3 modules: DSE, Temporal Feature Extractor, and a [CLS] classifier. The DSE module is designed to capture the collaborative and interactive spatial relationships within EEG signals evoked by emotional stimuli. Specifically, it transforms the raw EEG data into a compact and unified embedding representation. The Temporal Feature Extractor fully leverages the temporal information contained in the sequence of tokens produced by the DSE module. It comprises a preliminary processing layer, the pre-STM, followed by multiple stacked TAM layers that progressively refine the temporal representations. Finally, the [CLS] classifier utilizes the embedded [CLS] token to decode emotions, generating the final emotion classification output.

**Fig. 1.**
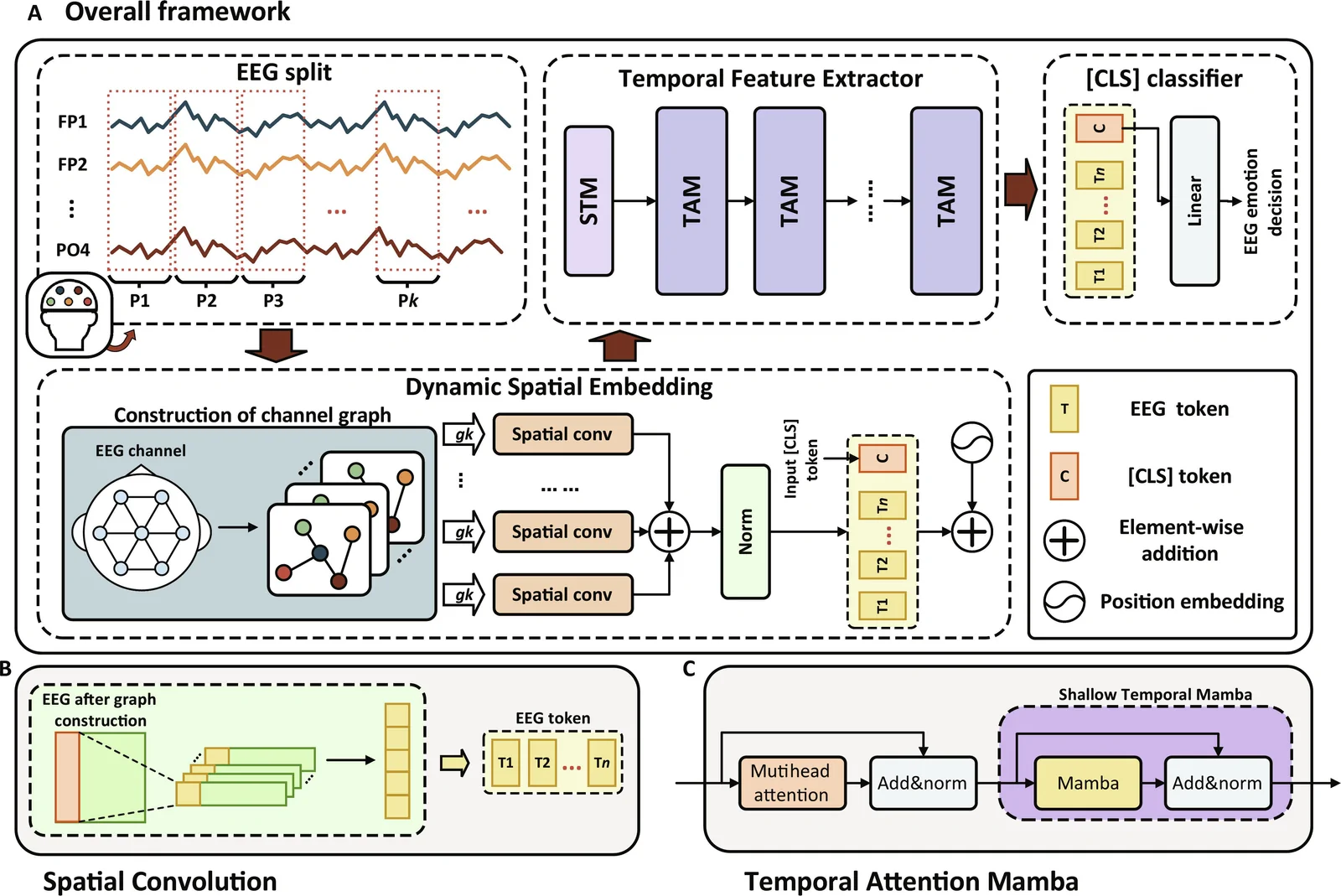
The framework of the Dynamic Spatial Embedding and Temporal Attention Mamba (DSE-TAM): (A) overall framework, (B) Spatial Convolution module, and (C) Temporal Attention Mamba module.

### Dynamic Spatial Embedding

To capture both local temporal dynamics and global spatial dependencies in EEG signals, we propose a DSE module that generates a unified embedding representation. The central design of DSE is to incorporate learnable graph construction into the EEG tokenization process. Specifically, the module first uses a trainable adjacency matrix to adaptively model the spatial dependencies among EEG electrodes. The resulting graph-filtered signals are not directly flattened for classification; instead, they are further projected into a sequence of compact token embeddings through convolution and pooling operations. Therefore, DSE serves as a graph-guided embedding layer that converts raw multichannel EEG signals into temporally ordered and spatially informed tokens. This design allows the subsequent Temporal Feature Extractor to model the evolution of brain-network-enhanced EEG representations over time.

Specifically, given raw EEG signals $X\in {\mathbb{R}}^{C\times T}$, where C is the number of EEG channels and T is the number of time points, the DSE module maps the input to an embedding space, yielding representations $T_{\mathrm{emb}}\in {\mathbb{R}}^{\left(J+1\right)\times D}$. Here, J denotes the number of temporal segments and D is the dimensionality of the learned embeddings.

To preserve the temporal continuity of EEG signals, the input EEG signal X is partitioned into J nonoverlapping segments, each containing P time points.

$$X=[X^{\left(1\right)},\;X^{\left(2\right)},\;\cdots X^{\left(J\right)}]$$ (1)

where $X^{\left(j\right)}\in {\mathbb{R}}^{C\times P}$ corresponds to the jth segment.

Within this graph-guided embedding process, EEG electrodes are treated as nodes in a graph $G=\left(V,\;E\right)$, where V and E represent the sets of nodes and edges, respectively. The spatial relationships among electrodes are encoded in an adjacency matrix $A\in {\mathbb{R}}^{C\times C}$. This matrix is initialized using Xavier initialization and subsequently treated as a learnable parameter during the training process, enabling the model to infer intrinsic inter-channel connectivity patterns in a data-driven manner. The normalized graph matrix L is then computed as

$$L=D^{-\frac{1}{2}}\left(A+A^{⊤}\right)D^{-\frac{1}{2}}$$ (2)

where D represents the degree matrix, with its diagonal elements defined as $D_{\mathrm{ii}}=\sum_{j}\left(A_{\mathrm{ij}}+A_{\mathrm{ji}}\right)$. This formulation not only guarantees numerical stability but also allows the spatial relationships between EEG electrodes to be dynamically adjusted throughout the training process.

A standard graph convolution based on the Laplacian ***L*** typically captures information only from immediate one-hop neighbors. However, brain activity involves dependencies across multiple spatial scales, requiring the integration of information from more distant nodes [24]. To capture such multiscale dependencies efficiently, we adopt spectral graph filters approximated by Chebyshev polynomials. This approach avoids the heavy cost of eigenvector decomposition and enables filters that flexibly adapt to neighborhoods of varying sizes through the polynomial order. In the DSE module, Chebyshev polynomial approximations of the spectral filter $M_{k}\left(L\right)$ are employed to extract multiscale spatial features. The polynomials are defined recursively as

$$M_{k}=\left\{\begin{array}{cc} I, & \text{if}\;k=0\\ L, & \text{if}\;k=1\\ 2{\mathrm{LM}}_{k-1}-M_{k-2}, & \text{if}\;k\geq 2 \end{array}\right.$$ (3)

where $M_{k}\left(L\right)$ represents the kth order polynomial. These polynomials allow the model to capture spatial dependencies at multiple scales.

Graph convolution is then applied to the segmented patches $X_{p}$, producing spatially filtered representations

$$\begin{array}{c} X_{g}^{k}=[M_{k}\left(L\right)X^{\left(1\right)}\;,M_{k}\left(L\right)X^{\left(2\right)}\;,\;\cdots \;,\;M_{k}\left(L\right)X^{\left(J\right)}] \end{array}$$ (4)

where $M_{k}\left(L\right)X^{\left(j\right)}$ represents the spatially filtered representation of the jth patch.

The filtered patches, $X_{g}^{k}$, are then processed by 2-dimensional convolutional layers to integrate spatial and temporal information. Each convolutional kernel has dimensions of $\left(C,\sqrt{P}\right)$, with a stride of $\sqrt{P}$, where *P* denotes the temporal length. The output of each convolution undergoes a Tanh activation, followed by power-average pooling over segments of $\sqrt{P}$ data points. These operations generate tokens $T_{g}^{k}\in {\mathbb{R}}^{J\times D}$, where *D* is the embedding dimension. The outputs from all *K* layers are then aggregated. Subsequently, batch normalization is applied to the aggregated output to produce the spatial–temporal embedding for each temporal segment. This process is mathematically expressed as

$$T_{g}=\mathrm{BN}\left(\sum_{k=0}^{K-1}\text{Pool}\left(\text{Tanh}\left(\text{Conv}\left(X_{g}^{k}\right)\right)\right)\right)$$ (5)

where $T_{g}\in {\mathbb{R}}^{J\times D}$ represents the spatial–temporal embedding for the *g*th temporal segment, capturing both local and global temporal–spatial features derived from the input patches.

To enhance the model’s capacity to capture temporal relationships across the sequence and construct a comprehensive sequence-level representation for emotion decoding [19], the process incorporates a learnable [CLS] token and positional encoding. The final embedding output from the DSE module is defined as

$$T_{\mathrm{emb}}=\left[t_{\mathrm{CLS}};\;t_{1};\;t_{2};\;\cdots ;\;t_{J}\right]+E_{\mathrm{pos}}$$ (6)

where $T_{\mathrm{emb}}\in {\mathbb{R}}^{\left(J+1\right)\times D}$ represents the resulting embedding, $t_{\mathrm{CLS}}\in {\mathbb{R}}^{D}$ is the learnable [CLS] token associated with the sequence-level representation, $t_{j}\in {\mathbb{R}}^{D}$ (j=1,2,…,J) denotes the embeddings for individual temporal segments, and $E_{\mathrm{pos}}\in {\mathbb{R}}^{\left(J+1\right)\times D}$ is the positional encoding matrix.

The positional encoding matrix $E_{\mathrm{pos}}$ is computed using the following formula for each position pos in the sequence:

$$\begin{array}{r} \;\mathrm{PE}\left(\mathrm{pos},\;2i\right)=\text{sin}\left(\frac{\mathrm{pos}}{10,000^{\frac{2i}{D}}}\right)\\ \mathrm{PE}\left(\mathrm{pos},\;2i+1\right)=\text{cos}\left(\frac{\mathrm{pos}}{10,000^{\frac{2i}{D}}}\right) \end{array}$$ (7)

where pos ranges from 1 to J+1, corresponding to the sequence after including the [CLS] token; D is the embedding dimension; and i indexes the dimensions within the embedding vector.

The unified representation captures both the spatial and temporal characteristics of EEG signals, serving as the input to subsequent modules for further processing.

### Temporal Feature Extractor

To effectively capture the temporal dynamics inherent in EEG signals, we reference prior work [13,15] by connecting the spatial and temporal modules in series. The Temporal Feature Extractor is designed as a hierarchical framework consisting of an initial STM module and multiple stacked TAM layers. Specifically, after the DSE module generates a sequence of spatially informed EEG tokens, an initial STM, denoted as pre-STM, is first applied to provide lightweight temporal preprocessing and refine the token sequence before deeper temporal modeling. The processed sequence is then fed into the stacked TAM layers, where each TAM layer contains a self-attention submodule followed by an STM submodule. In this way, the overall temporal extractor can be summarized as Temporal Feature Extractor = pre-STM + *L* × TAM(STA + STM). Here, the pre-STM and the STM inside each TAM layer share the same basic Mamba-based block design, but they play different roles: the former prepares the embedded EEG tokens for subsequent temporal modeling, whereas the latter refines temporal dynamics within each TAM layer. This structured hybrid approach progressively models both short-term fluctuations and long-range dependencies, thereby enhancing the model’s capacity to represent EEG dynamics.

#### Shallow Temporal Mamba

We incorporate the selective SSM (Mamba) into our framework to effectively capture long-term temporal dependencies in the embedded EEG tokens. Mamba selectively emphasizes relevant information while filtering out noise [18]. The architecture consists of 2 distinct branches, $B_{1}\left(\cdot \right)$ and $B_{2}\left(\cdot \right)$, which process the embedded temporal features $T_{\mathrm{emb}}$ in complementary ways. The mathematical formulation for the 2 branches and the final Mamba output is

$$\begin{array}{c} B_{1}\left(T_{\mathrm{emb}}\right)=\mathrm{SSM}\left(\sigma \left(\text{Conv}1D\left(\text{Linear}\left(T_{\mathrm{emb}}\right)\right)\right)\right) \end{array}$$ (8)

$$B_{2}\left(T_{\mathrm{emb}}\right)=\sigma \left(\text{Linear}\left(T_{\mathrm{emb}}\right)\right)$$ (9)

$$\begin{array}{c} \text{Mamba}\left(T_{\mathrm{emb}}\right)=\text{Linear}\left(B_{1}\left(T_{\mathrm{emb}}\right)⊙B_{2}\left(T_{\mathrm{emb}}\right)\right) \end{array}$$ (10)

Here, $\text{Linear}\left(\cdot \right)$ represents a linear projection layer, while $\text{Conv}1D\left(\cdot \right)$ denotes a one-dimensional convolutional layer. The function $\sigma \left(\cdot \right)$ is the SiLU/Swish activation function, as described in Refs. [25,26]. The selective SSM is represented by $\mathrm{SSM}\left(\cdot \right)$ [27]. The symbol ⊙ indicates a nonlinear operation, which may be an activation function or element-wise multiplication. The Mamba block output, $\text{Mamba}\left(T_{\mathrm{emb}}\right)$, preserves the dimensionality of the input embedded temporal features, $T_{\mathrm{emb}}$.

To efficiently capture EEG-specific temporal dependencies, we propose the STM module, a lightweight yet expressive component built upon the Mamba architecture. STM leverages Mamba’s dynamic temporal modeling capabilities, incorporating residual connections and layer normalization to promote stable training and improved performance. Specifically, the input embedding, denoted by $T_{\mathrm{emb}}$, undergoes processing by the Mamba mechanism to extract relevant temporal features. These features are then combined with the original input via a residual connection, followed by layer normalization and dropout regularization to mitigate overfitting. The complete STM operation is mathematically expressed as

$$\mathrm{STM}\left(T_{\mathrm{emb}}\right)=\text{Norm}\left(T_{\mathrm{emb}}+\text{Drop}\left(\text{Mamba}\left(T_{\mathrm{emb}}\right)\right)\right)$$ (11)

STM initially serves as a preprocessing layer, transforming the embedded input sequence into a form suitable for further temporal feature extraction. This process is formulated as $T_{0}=\mathrm{STM}\left(T_{\mathrm{emb}}\right)$, where $T_{0}$ serves as the input to the subsequent primary temporal feature extraction modules, ensuring effective preparation of the sequence for global modeling.

#### Temporal Attention Mamba

The TAM module employs a hierarchical architecture combining self-attention and SSMs to capture both global and local temporal dependencies in EEG signals. By interleaving submodules, TAM progressively refines temporal features, balancing coarse-grained global relationships with fine-grained local dynamics for effective EEG representation. Specifically, TAM processes an input embedding $T_{0}$ through a series of layers, each comprising 2 distinct submodules. The initial submodule utilizes a multihead self-attention (STA) mechanism to model long-range temporal dependencies across the entire time series.

The self-attention mechanism starts by projecting the input $T_{0}\in {\mathbb{R}}^{\left(N+1\right)\times D}$ into 3 matrices, query (Q), key (K), and value (V), through learned linear transformations:

$$Q=T_{0}W_{Q},\;K=T_{0}W_{K},\;V=T_{0}W_{V}$$ (12)

where $W_{Q},W_{K},W_{V}\in {\mathbb{R}}^{D\times d}$ are trainable weight matrices and d is the dimensionality of each attention head.

The attention scores are computed using scaled dot-product attention:

$$\begin{array}{c} \text{Attention}\left(Q,K,V\right)=\text{Softmax}\left(\frac{QK^{⊤}}{\sqrt{d}}\right)V \end{array}$$ (13)

where $1/\sqrt{d}$ is a scaling factor to prevent the dot products from becoming too large, stabilizing the gradients during training.

For multihead attention, the input is split into H heads, and the attention operation is performed in parallel. The outputs of all heads are concatenated and projected back to the original dimension:

$$\begin{array}{c} \text{MultiHead}\left(Q,K,V\right)=\text{Concat}\left({\text{head}}_{1},\ldots ,{\text{head}}_{H}\right)W_{O} \end{array}$$ (14)

where $W_{O}\in {\mathbb{R}}^{\mathrm{Hd}\times D}$ is the output projection matrix.

The output of the attention mechanism is stabilized using residual connections, layer normalization, and dropout. This yields the self-attention submodule’s final output:

$$\begin{array}{c} \mathrm{STA}\left(T_{0}\right)=\text{Norm}\left(T_{0}+\text{Drop}\left(\text{MultiHead}\left(Q,K,V\right)\right)\right) \end{array}$$ (15)

Following the self-attention mechanism, the output $\mathrm{STA}\left(\cdot \right)$ is passed to the second submodule, which leverages the $\mathrm{STM}\left(\cdot \right)$ mechanism, as previously defined in Eq. (11), for dynamic temporal modeling. This submodule captures intricate temporal dependencies by dynamically parameterizing state-space transitions, thereby enhancing the representation of localized dynamics in the EEG signals. The hybrid structure of TAM is defined as

$$\mathrm{TAM}\left(T_{0}\right)=\mathrm{STM}\left(\mathrm{STA}\left(T_{0}\right)\right)$$ (16)

where the interplay between $\mathrm{STA}\left(\cdot \right)$ and $\mathrm{STM}\left(\cdot \right)$ enables TAM to effectively integrate global and local temporal features, making it well suited for modeling the complex temporal dynamics inherent in EEG signals.

By stacking L such layers and connecting them with the DSE module in series, TAM alternates between global and local modeling to form a hierarchical structure. Each layer incrementally refines the temporal patterns learned from the previous one, enabling the extraction of both high-level abstractions and fine-grained details. The final output of the TAM module is obtained by applying dropout to the output of the last layer, enhancing generalization and preventing overfitting:

$$T_{L}=\text{Drop}\left({\mathrm{TAM}}_{L}\left(\ldots \left({\mathrm{TAM}}_{1}\left(T_{0}\right)\right)\right)\right)$$ (17)

### [CLS] token classifier

For classification, the [CLS] token’s contextualized representation is fed into a fully connected layer followed by a softmax activation function to predict the input data labels. Specifically, the predicted label vector $\hat{y}$ is obtained by processing the [CLS] token representation, $t_{\mathrm{CLS}}$, as follows:

$$\hat{y}=\mathrm{CLS}\left(t_{\mathrm{CLS}}\right)\in {\mathbb{R}}^{N_{c}}$$ (18)

Here, $t_{\mathrm{CLS}}$ represents the learned [CLS] token embedding, derived from the output embeddings $T_{L}$ of the preceding transformer layers. $N_{c}$ denotes the number of classes.

To train the classifier, we employ the cross-entropy (CE) loss function, which quantifies the discrepancy between the predicted label distribution $\hat{y}$ and the ground truth labels y.

$$\begin{array}{c} L_{c}=\mathrm{CE}\left(\hat{y}\right)=-\frac{1}{M}\sum_{i=1}^{M}\sum_{c=1}^{N_{c}}y_{\mathrm{ic}}\;\text{log}\left({\hat{y}}_{\mathrm{ic}}\right) \end{array}$$ (19)

where M is the number of training samples and $y_{\mathrm{ic}}$ represents the ground truth label for the ith sample and the cth class.

### Experiments

#### Datasets

To comprehensively evaluate the proposed model and in line with extensive prior work [24,28], we conducted experiments across both dimensional (valence and arousal) and categorical (e.g., happy and sad) emotion representations. The evaluation was performed on 3 datasets: the publicly available DEAP dataset [29], the DREAMER dataset [30], and CEED, a dataset collected in our laboratory.

The DEAP dataset consists of multimodal recordings from 32 participants, including 32-channel EEG signals and peripheral physiological signals, collected during emotion elicitation via music video stimuli. Participants watched 40 music video clips, each designed to evoke different emotional states. For each trial, a 60-s preprocessed EEG segment is recorded and downsampled to 128 Hz. To mitigate artifacts, such as ocular movements, a combination of blind source separation and band-pass filtering (4 to 45 Hz) is applied. Participants rated their emotional states in terms of valence, arousal, dominance, and liking using a 9-point scale.

The DREAMER dataset includes both EEG and ECG signals, recorded during affect elicitation through audiovisual stimuli. EEG signals from 23 participants (14 males and 9 females) were captured using an Emotive EPOC system with 14 EEG channels at a sampling rate of 128 Hz. Each participant viewed 18 film clips with durations ranging from 65 to 393 s, which were specifically selected to elicit distinct emotional states. Emotional annotations for each clip were gathered using a 5-level scale for valence, arousal, and dominance. In addition to the EEG recordings, 4 s of baseline data were recorded before each clip.

The CEED dataset captures emotional responses to video stimuli within a naturalistic paradigm. The study was approved by the Chongqing University of Posts and Telecommunications, and all participants provided written informed consent. Unlike DEAP and DREAMER, which use dimensional labeling, CEED adopts a categorical scheme classifying emotions as happy, neutral, or sad. The dataset includes EEG recordings from 35 participants (mean age 22 years). Stimulus materials comprised 15 Chinese video clips (5 per valence category), selected for short duration, comprehensibility without explanation, and reliable emotion elicitation. Each clip lasted approximately 3 min. During the experiment, participants pressed a key to start after reading instructions. Each block began with a 5-s cue, followed by a video clip and a 15-s rest. After all clips, participants reported their emotional responses via questionnaire. Before the experiment, participants’ psychological state was assessed, and they were instructed to remain calm and well rested. The experiment was conducted in a dimly lit, comfortable laboratory. EEG data were collected using a 64-channel Brain Products system (63 active electrodes, excluding reference), amplified with an actiCHamp Plus amplifier, and positioned according to the standard 10–20 system.

Consistent with prior studies [15,31], baseline drift was corrected by subtracting the mean baseline signal from each trial’s stimulus-related EEG segment. A standardized preprocessing pipeline was applied to all 3 datasets: data were downsampled to 128 Hz and segmented into nonoverlapping 3-s epochs. The resulting data tensors were of the following dimensions: DEAP ($X\in {\mathbb{R}}^{800\times 32\times 384}$), DREAMER ($X\in {\mathbb{R}}^{1,242\times 14\times 384}$), and CEED ($X\in {\mathbb{R}}^{955\times 63\times 384}$).

#### Benchmark algorithms

The following 7 methods were employed as the benchmark algorithms:

• Dynamical graph convolutional neural network (DGCNN) [24], which is a dynamic GCN that automatically learns inter-channel relationships through trainable adjacency matrices, eliminating the need for pre-defined neurophysiological connectivity patterns.
• Attention-based convolutional recurrent neural network (ACRNN) [20], which leverages convolutional layers for spatial feature extraction and long short-term memory networks to model temporal dependencies, augmented by channel-wise and temporal attention mechanisms to emphasize discriminative features.
• EEG Conformer [13], which combines CNNs for local feature extraction with transformers to capture global temporal correlations
• TSception [32], which is a multiscale CNN architecture designed to extract dynamic temporal features and spatial patterns through asymmetric spatial layers and advanced fusion techniques, effectively enhancing emotion recognition accuracy.
• LGGNet [33], which is a neurologically inspired graph neural network (GNN) that simultaneously models local and global brain connectivity patterns through learnable graph filters. It integrates multiscale temporal convolutions, attentive fusion, and graph-filtering layers to capture complex spatial–temporal patterns.
• EEG-Deformer [14], which is a dense convolutional transformer incorporating a Fine-grained Temporal Learning branch into transformer for capturing coarse-to-fine temporal patterns and a Dense Information Purification module for adaptively fusing and refining multi-level temporal features.
• miMamba [28], which leverages a Mamba structure to efficiently model the interactions between dynamic temporal dependencies and spatial characteristics while also capturing multiscale temporal features. (The implementation of this model was reproduced by ourselves based on the original study.)

To ensure consistency and fairness, all benchmark methods were implemented with unified training settings and evaluation protocols. For input representations, we followed the original design of each baseline model. DGCNN was originally formulated for electrode-level DE features, and directly applying it to raw EEG signals would require additional temporal embedding or architectural modification. Therefore, DGCNN was evaluated using DE features, whereas the other baseline methods, which are designed for raw EEG decoding, were evaluated using raw EEG signals. Under this protocol, each method was compared using its architecture-compatible input setting, while the data split, training strategy, and evaluation metrics were kept consistent across all models.

#### Experiment protocol

The performance of the proposed model was assessed using 2 kinds of experiments, i.e., trial-wise *K*-fold cross-validation and leave-one-subject-out (LOSO). Statistical significance was established via a Wilcoxon signed-rank test, with *P* values adjusted for multiple comparisons using the false discovery rate procedure.

For the trial-wise *K*-fold cross-validation experiment, data were partitioned into *K* folds along the trials, ensuring that segments from a single trial remained within the same fold. In each iteration, one fold served as the testing set, and the remaining *K* folds constituted the training set. Recognizing the varying number of trials across datasets, the value of *K* was selected to maintain a balanced number of trials per fold while adhering to a testing data proportion between 10% and 20%. Specifically, a 10-fold cross-validation was implemented for the DEAP dataset (40 trials), a 6-fold cross-validation for the DREAMER dataset (18 trials), and a 5-fold cross-validation for the CEED dataset (15 trials). This approach optimized the trade-off between maximizing training data volume and maintaining a sufficient testing data proportion for robust performance evaluation.

To evaluate the model’s generalization capability to new subjects, we adopted a LOSO cross-validation strategy. In each iteration, data from one subject were held out for testing, while the remaining subjects’ data were used for training. This evaluation ensured that the model’s performance was assessed on completely independent data, providing a robust estimate of its ability to generalize to new, unseen individuals.

#### Implementation details

Our method is implemented using the PyTorch library in Python 3.10, running on a machine equipped with an NVIDIA GeForce RTX 3090 graphics processing unit. The model is trained using the RMSprop optimizer with an initial learning rate of 1 × 10^−3^, a weight decay of 1 × 10^−6^, and an alpha parameter of 0.99 to control the moving average of squared gradients. The maximum number of epochs is set to 500, and a batch size of 256 is used to ensure efficient optimization and convergence. The computational complexity analysis is provided in the Supplementary Materials.

For the network parameter configuration, all hyperparameters are kept consistent across all datasets, with the only adjustment being the number of EEG channels according to each dataset’s input specification. The model includes 4 graph convolutional layers. Each EEG token encompasses 16 time points, and with EEG signals sampled at 128 Hz over a 3-s segment, the total signal length is 384 points. The token embedding dimension is set to 48. The entire Temporal Feature Extractor incorporates with 2 layers of TAM. Furthermore, it is important to note the training strategy for the learnable adjacency matrix ***A*** within the DSE module. In our implementation, ***A*** is trained as a global, shared parameter across all subjects within a given dataset. This approach enables the model to learn a generalized spatial relationship common to the population, rather than a subject-specific one, thereby enhancing the model’s generalization capabilities. The source code of the proposed model is available at https://github.com/Raizeroko/DSE-TAM.

## Results

### Decoding accuracies

Table 1 presents the results of trial-wise *K*-fold cross-validation, demonstrating that DSE-TAM consistently achieves superior performance compared to existing methods across all datasets and evaluation metrics. Specifically, for arousal decoding on the DEAP dataset, DSE-TAM attained an accuracy of 79.75%, surpassing Conformer (78.32%) and exhibiting statistically significant improvement over other baseline methods (P<0.05). In valence decoding on DEAP, DSE-TAM reached an accuracy of 78.27%, a significant improvement over that of Deformer (75.71%, P<0.05). On the DREAMER dataset, DSE-TAM achieved an accuracy of 80.55% in arousal decoding, significantly outperforming LGGNet (79.08%, P<0.05). Furthermore, DSE-TAM achieved a leading accuracy of 83.91% in valence decoding on DREAMER, representing a statistically significant 3.15% improvement over LGGNet (P<0.05). For the 3-class emotion recognition task on the CEED dataset, DSE-TAM achieved an accuracy of 73.75%, outperforming DGCNN, ACRNN, and LGGNet while exhibiting comparable performance to TSception and Conformer. These results highlight the robust generalization capability and adaptability of DSE-TAM across a diverse range of emotion decoding tasks.

**Table 1.** Model performance using trial-wise *K*-fold cross-validation. * indicates *P* < 0.05, ** indicates *P* < 0.01, and *** indicates *P* < 0.001. Boldface indicates the best performance for each dataset and metric.

Dataset	Metric	DGCNN/%	ACRNN/%	TSception/%	Conformer/%	LGGNet/%	Deformer/%	miMamba/%	Ours/%
DEAP	Arousal	73.54 ± 5.22***	68.79 ± 12.32***	75.41 ± 6.01**	78.32 ± 4.98	77.14 ± 6.04*	77.80 ± 4.86*	74.78 ± 7.38***	**79.75 ± 3.61**
	Valence	74.34 ± 6.00**	65.84 ± 12.98***	74.85 ± 4.34**	75.59 ± 3.10**	73.84 ± 4.41***	75.71 ± 5.14*	70.75 ± 4.73***	**78.27 ± 5.01**
DREAMER	Arousal	73.02 ± 6.29***	72.20 ± 7.33***	77.45 ± 7.36*	73.03 ± 9.21**	79.08 ± 6.21	75.21 ± 7.21**	73.67 ± 6.02***	**80.55 ± 6.68**
	Valence	71.32 ± 9.54***	75.58 ± 6.56***	77.79 ± 9.28***	76.33 ± 7.01***	80.76 ± 7.43*	76.18 ± 6.42***	75.19 ± 5.72***	**83.91 ± 7.07**
CEED	H/N/S	60.87 ± 10.48***	41.58 ± 14.72***	**74.72 ± 11.40**	74.12 ± 8.92	67.48 ± 9.11***	71.66 ± 10.33*	55.21 ± 5.08***	73.75 ± 10.04

DGCNN, dynamical graph convolutional neural network, ACRNN, attention-based convolutional recurrent neural network; H/N/S, happy/neutral/sad

The robustness of the proposed model was evaluated using LOSO cross-validation. The corresponding results, detailed in Table 2, demonstrate the model’s ability to generalize to unseen subjects. Specifically, for the DEAP dataset, the model attained classification accuracies of 61.29% and 58.80% for arousal and valence, respectively, which remain competitive and outperform ACRNN and DGCNN. On the DREAMER dataset, DSE-TAM achieved classification accuracies of 61.96% for arousal and 65.11% for valence, surpassing all benchmark methods. For the CEED dataset, DSE-TAM achieved a classification accuracy of 59.59% for the 3-class emotion task. Although the model slightly lags behind TSception and Conformer under *K*-fold validation on CEED, it demonstrates superior performance in the more challenging LOSO cross-validation setting. Moreover, compared with miMamba, which also employs the Mamba architecture, our model achieves significantly superior performance (P<0.001) under both cross-validation protocols. Overall, these findings indicate that DSE-TAM maintains consistent and competitive performance across diverse datasets and validation paradigms.

**Table 2.** Model performance using LOSO cross-validation. * indicates *P* < 0.05, ** indicates *P* < 0.01, and *** indicates *P* < 0.001. Boldface indicates the best performance for each dataset and metric.

Dataset	Metric	DGCNN/%	ACRNN/%	TSception/%	Conformer/%	LGGNet/%	Deformer/%	miMamba/%	Ours/%
DEAP	Arousal	57.12 ± 9.81	57.73 ± 15.62**	59.76 ± 10.58	**62.30 ± 11.80**	60.84 ± 7.94	60.36 ± 9.93	52.77 ± 6.53***	61.29 ± 12.54
	Valence	56.63 ± 8.95	55.31 ± 9.14***	55.39 ± 7.78	58.51 ± 5.65	58.25 ± 4.30	57.75 ± 4.58	50.22 ± 3.11***	**58.80 ± 6.90**
DREAMER	Arousal	57.93 ± 8.17	58.75 ± 7.21***	58.73 ± 6.81	59.41 ± 6.64	59.92 ± 8.83	59.57 ± 8.14	49.94 ± 6.46***	**61.96 ± 5.85**
	Valence	61.26 ± 7.29	61.58 ± 7.19**	64.60 ± 6.68	63.46 ± 8.29	63.63 ± 7.80	64.43 ± 8.12	45.86 ± 5.00***	**65.11 ± 7.21**
CEED	H/N/S	56.81 ± 8.30	58.23 ± 15.37	58.08 ± 9.91	59.32 ± 9.24	59.00 ± 8.70	58.86 ± 9.68	33.91 ± 2.53***	**59.59 ± 9.75**

LOSO, leave-one-subject-out; DGCNN, dynamical graph convolutional neural network, ACRNN, attention-based convolutional recurrent neural network; H/N/S, happy/neutral/sad

A deeper analysis suggests the baselines’ varied performance stems from their architectural trade-offs. For instance, the reliance of models like Conformer and Deformer on standard CNNs for spatial learning imposes a key limitation: their fixed, local receptive fields are less adept at capturing the brain’s dynamic, nonlocal connectivity compared to our adaptive DSE module. Similarly, TSception’s success on the high-density CEED dataset can be attributed to its specialized multiscale convolutions, but this fixed-scale approach appears less effective on sparser datasets where modeling global relationships is more critical. In essence, while GNN-based models like DGCNN and LGGNet focus on spatial aspects, they may not capture temporal dynamics as effectively as our TAM module. miMamba relies on a pure Mamba architecture, which lacks an effective mechanism for capturing global spatial dependencies. Consequently, it struggles to effectively model complex long-term emotion representations. The baselines often exhibit constraints in either their spatial or temporal modeling, whereas DSE-TAM’s synergistic design excels by adaptively capturing complex dynamics in both domains.

### Confusion metrics

Fig. 2 shows the confusion matrices on 3 datasets. The classification results on the DEAP dataset are illustrated in the first and second rows of Fig. 2. Baseline models generally show a bias toward classifying high-valence and high-arousal states more accurately. In contrast, our proposed DSE-TAM demonstrates that a more balanced classification performance achieves a substantial performance gain in the low-arousal category. The third and fourth rows of Fig. 2 detail the results obtained using the DREAMER dataset. The performance trend of baseline models shifts, showing a preference for low-arousal and low-valence classification. DSE-TAM again exhibits robust and balanced capabilities, achieving substantial improvements in the high-valence category and maintaining high accuracy (92.03%) for low-valence states. For the CEED dataset, our method demonstrates enhanced discrimination between sad and neutral emotions compared to existing approaches. Although performance on positive emotion classification is comparatively lower, the overall results confirm DSE-TAM’s efficacy in discerning subtle emotional states and its superior ability to differentiate between similar emotional categories.

**Fig. 2.**
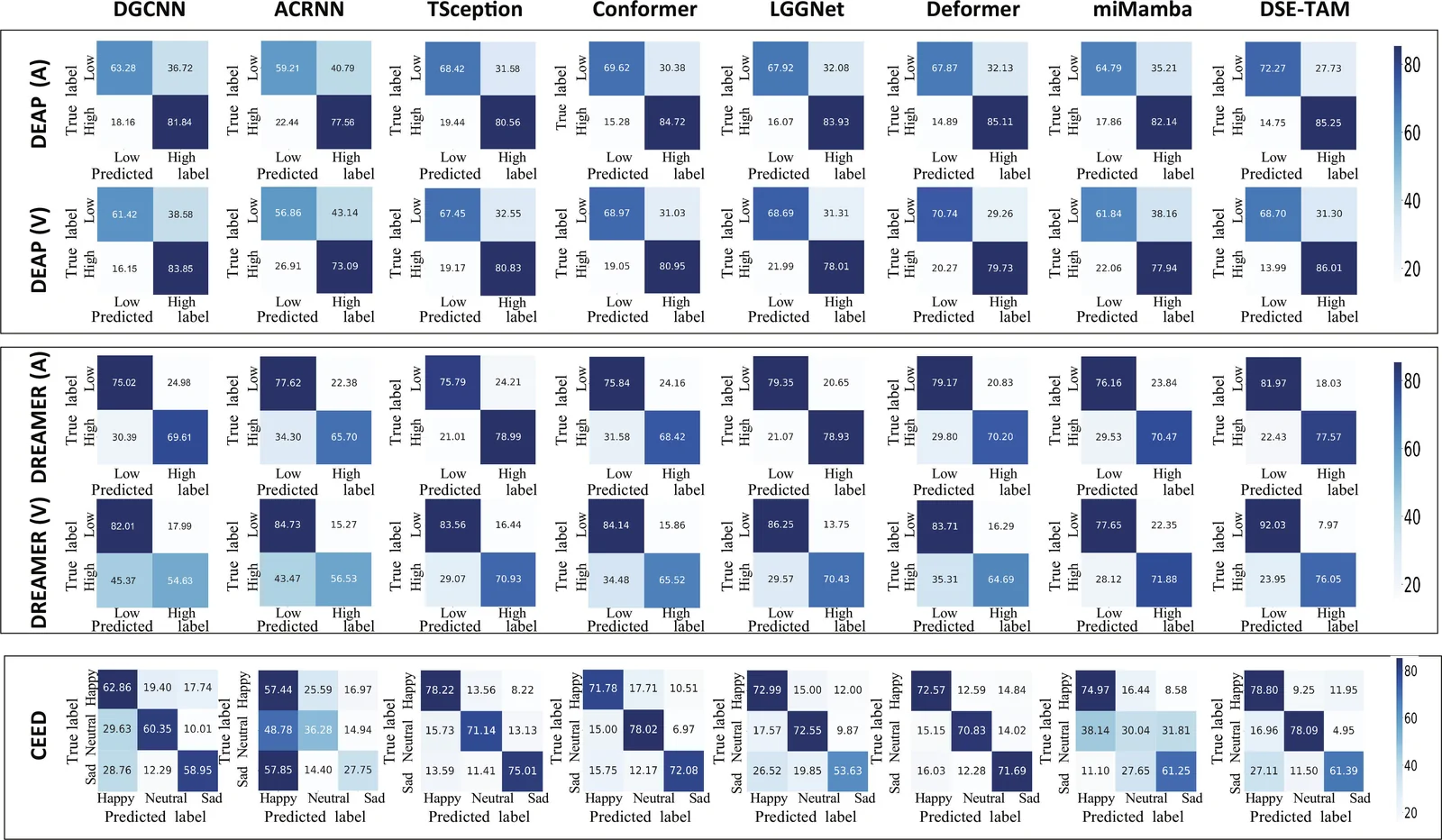
Valence and arousal confusion matrices for the DEAP, DREAMER, and CEED datasets. Columns represent model performance: dynamical graph convolutional neural network (DGCNN), attention-based convolutional recurrent neural network (ACRNN), TSception, Conformer, LGGNet, Deformer, miMamba and Dynamic Spatial Embedding and Temporal Attention Mamba (DSE-TAM; ours).

In summary, our proposed DSE-TAM method consistently demonstrates a key advantage: it substantially improves the classification accuracy of challenging emotional states, specifically those with low arousal and low valence, while also achieving superior classification performance on negative and neutral states, thereby validating its capacity to model complex spatiotemporal relationships and enhance the decoding of challenging emotional states.

### Ablation study

To evaluate the contribution of each module in our model, we performed ablation experiments by systematically removing or modifying key components and analyzing their impact on performance. Specifically, we assessed the model’s performance under the following conditions: removing the [CLS] token and using the sequence of output tokens as input to the classifier, excluding the DSE module, and removing the pre-STM layer before feeding the input into the multilayer TAM. Additionally, we investigated the effects of replacing all STM modules with STA modules, as well as replacing all STA modules within the model with STM modules. The ablation experiments were conducted on the DEAP, DREAMER, and CEED datasets using trial-wise *K*-fold cross-validation, and the results are summarized in Table 3.

**Table 3.** Ablation study on DEAP, DREAMER, and CEED using trial-wise *K*-fold validation. * indicates *P* < 0.05, ** indicates *P* < 0.01, and *** indicates *P* < 0.001.

Dataset	Method	Arousal/%	Valence/%	H/N/S/%
DEAP	W/o [CLS]	75.67 ± 5.04**	72.47 ± 4.85***	ND
	W/o DSE	73.65 ± 4.42***	71.39 ± 4.69***	ND
	W/o pre-STM	76.00 ± 4.27**	74.37 ± 3.65**	ND
	W/o STM	75.84 ± 4.59**	74.62 ± 4.15**	ND
	W/o STA	68.20 ± 9.09***	59.53 ± 8.56***	ND
	DSE-TAM	79.75 ± 3.61	78.27 ± 5.01	ND
DREAMER	W/o [CLS]	78.68 ± 5.23	79.86 ± 5.63**	ND
	W/o DSE	78.91 ± 7.37	83.74 ± 7.09	ND
	W/o pre-STM	76.98 ± 7.40**	80.13 ± 7.94**	ND
	W/o STM	74.29 ± 5.89**	75.66 ± 8.05*	ND
	W/o STA	63.28 ± 12.24***	63.63 ± 12.23***	ND
	DSE-TAM	80.55 ± 6.68	83.91 ± 7.07	ND
CEED	W/o [CLS]	ND	ND	65.03 ± 10.40***
	W/o DSE	ND	ND	70.89 ± 9.63*
	W/o pre-STM	ND	ND	71.74 ± 9.74
	W/o STM	ND	ND	61.00 ± 12.01***
	W/o STA	ND	ND	44.43 ± 3.40***
	DSE-TAM	ND	ND	73.75 ± 10.04

H/N/S, happy/neutral/sad; DSE, Dynamic Spatial Embedding; STM, Shallow Temporal Mamba; DSE-TAM, Dynamic Spatial Embedding and Temporal Attention Mamba; ND, no data/not applicable

The results, presented in Table 3, reveal a consistent trend across all 3 datasets, underscoring the indispensable role of each component. The most pronounced performance degradation occurred upon removing the STA modules, which resulted in accuracy drops of up to 18.67% on DEAP, 20.28% on DREAMER, and 29.32% on CEED. This highlights a critical synergy: relying solely on STM’s forward state transitions severely hinders the [CLS] token, placed at the sequence’s start, from effectively aggregating information from the entire sequence. Similarly, other architectural modifications—such as removing the STM, DSE, or [CLS] token—consistently led to substantial performance reductions. These outcomes reaffirm the complementary nature of STM and STA, the necessity of DSE for capturing spatial relationships, and the critical function of the [CLS] token in consolidating global contextual information. These comprehensive findings validate the efficacy and robust design of the proposed integrated framework.

## Discussion

### Effects of EEG token encompassing temporal length *P*

The token encompassing temporal length, denoted as *P*, is a crucial hyperparameter in our model’s architecture. It dictates the temporal receptive field of the initial tokenization stage and subsequently influences the dimensions of the 2-dimensional convolutional kernels and pooling operations, as detailed in Eq. (5). To ascertain the optimal value for *P*, we conducted a comprehensive sensitivity analysis across our target datasets.

We evaluated the model’s performance across *P* values of {4, 8, 16, 32, 64}, where convolutional kernel and pooling dimensions were set symmetrically to $\sqrt{P}$ (e.g., 4 × 4 for *P* = 16) or as factor pairs for nonsquare values (e.g., 4 × 8 for *P* = 32). As illustrated in Fig. 3, the analysis clearly identifies *P* = 16 as the optimal choice, yielding consistently superior performance. Although a configuration from *P* = 32 was optimal in the singular case of the DREAMER-valence task, the *P* = 16 setting remained highly competitive as the second best performer, reinforcing its selection for robust, generalized performance.

**Fig. 3.**
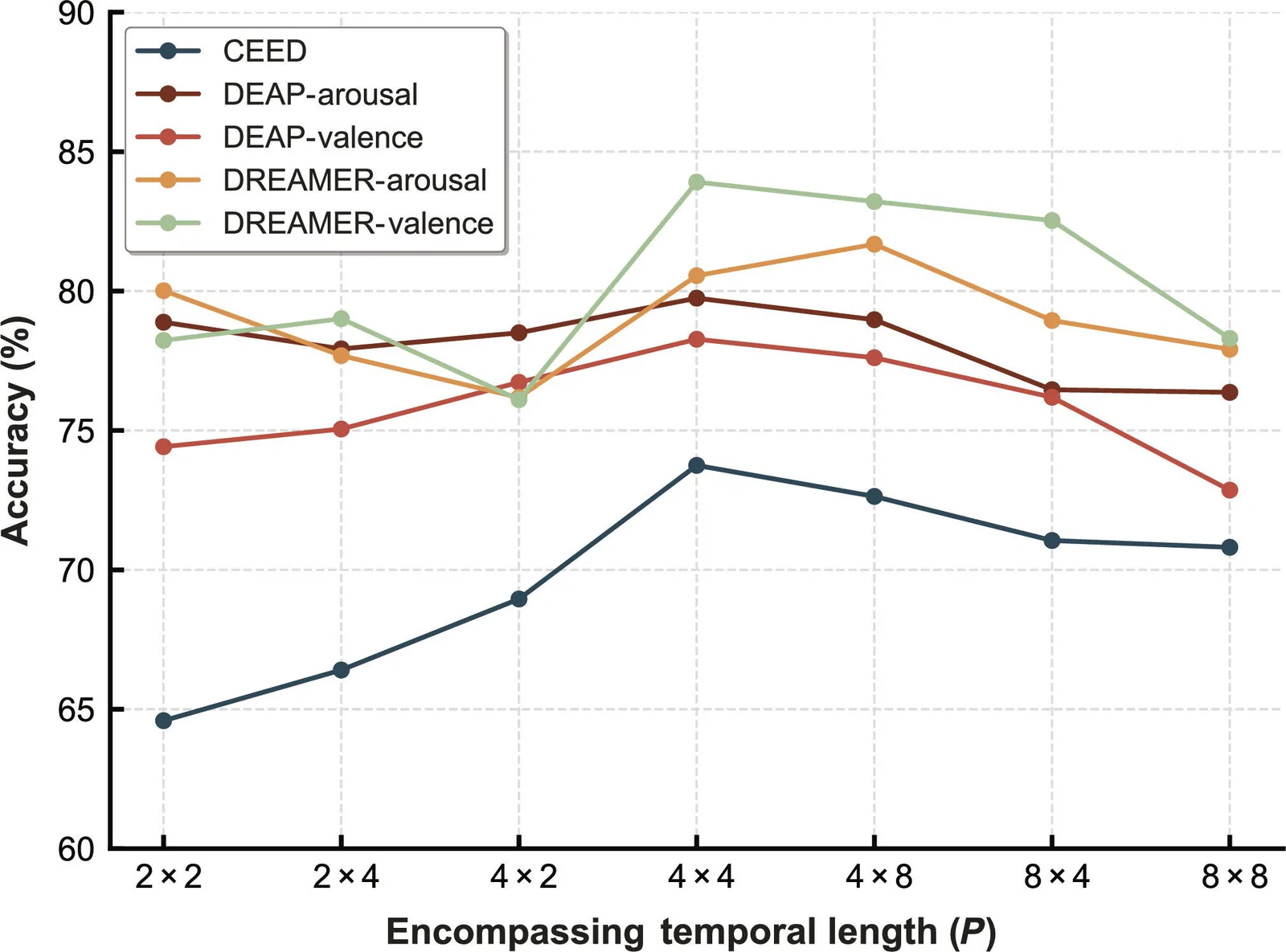
Performance sensitivity to the token encompassing temporal length (*P*). Each curve represents the model’s performance on a different dataset and affective dimension.

Therefore, considering its robust and superior performance across the vast majority of experimental settings, we selected *P* = 16 as the definitive value for our architecture. This choice provides the best trade-off between performance and generalizability.

### Impact of the [CLS] token position

In our architecture, the [CLS] token functions as a dedicated sequence-level representation, evolving during training into an optimized “emotional aggregator” [34]. Following prevalent methodologies [35–37], we prepended the [CLS] token to the EEG sequence but also conducted experiments to evaluate the performance sensitivity to its placement. We systematically varied its position to the beginning, middle, and end of the sequence. As depicted in Fig. 4, placing the [CLS] token at the beginning consistently yields superior performance across all datasets.

**Fig. 4.**
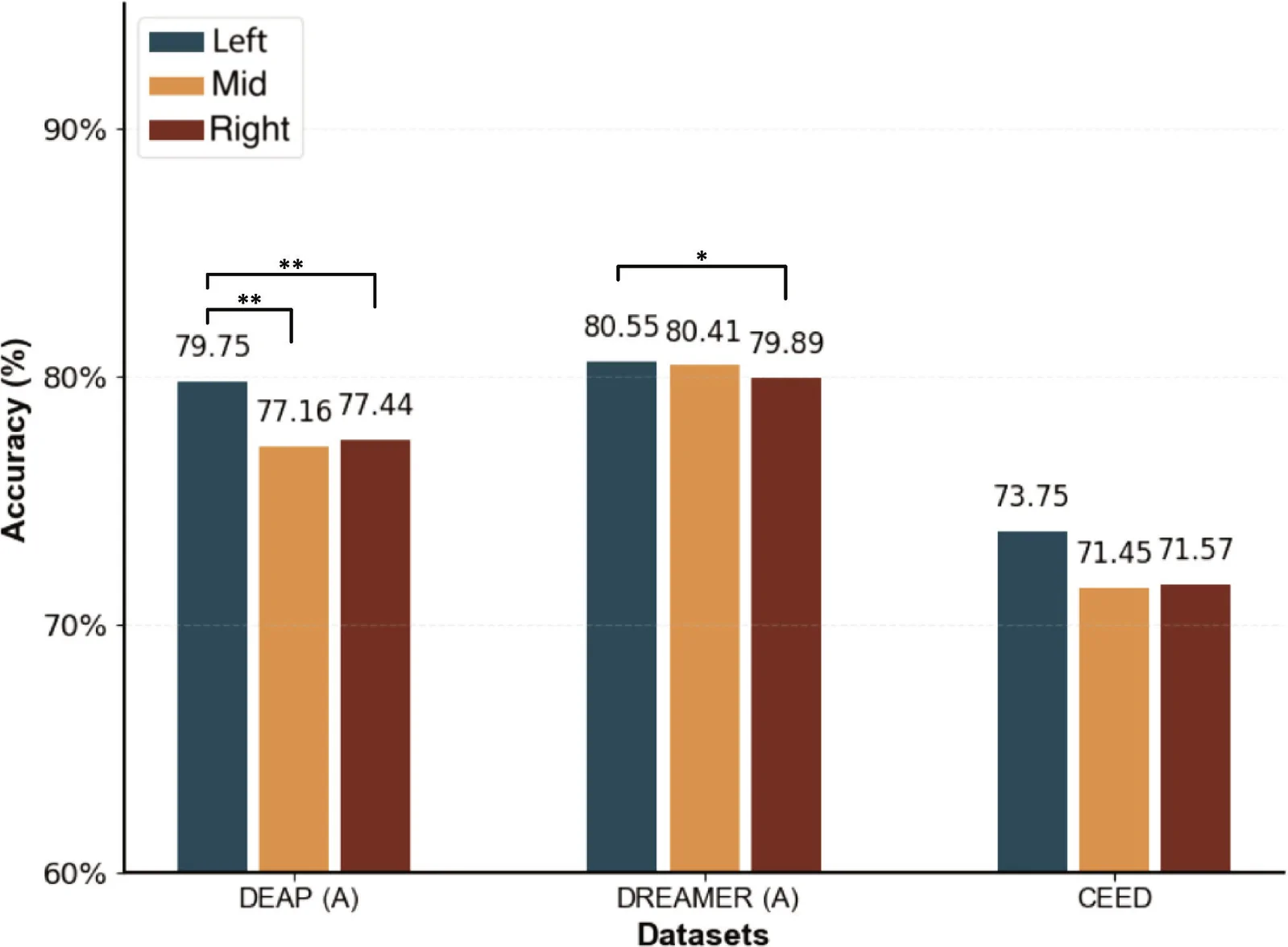
Impact of the [CLS] token position on emotion decoding performance. The figure compares performance across 3 datasets for left, middle, and right [CLS] token positions (arranged in left-to-right order). “*” indicates *P* < 0.05, and “**” indicates *P* < 0.01.

Placing the token in the middle or at the end of the sequence leads to a notable degradation in performance. A middle placement appears to hinder the token’s ability to form a holistic representation of the entire EEG sequence. An end placement is likely hampered by the accumulated state dependencies of the sequential model, which can suppress the contribution of earlier features and potentially introduce gradient conflicts during back-propagation. These empirical findings underscore the importance of the leftmost [CLS] token placement for robust emotion decoding.

The superiority of this leftmost placement is particularly synergistic with our DSE-TAM framework. Positioned at the beginning, the [CLS] token effectively leverages both the sequential state update mechanism of Mamba and the global attention connections inherent in TAM. This strategic placement allows it to serve as a robust anchor, progressively aggregating sequence-level information while capturing the full temporal dynamics of the EEG data, thus facilitating an optimal integration of spatial and temporal features for emotion decoding.

### Temporal feature extraction structures

To investigate the impact of diverse temporal modeling approaches on EEG-based emotion recognition, we evaluated 6 architectures integrating the STM, STA, and Feed-Forward Network (FFN) modules. The performance results, summarized in Table 4, demonstrate that our proposed DSE-TAM consistently achieves the most robust and accurate results across all datasets.

**Table 4.** Comparison of different temporal feature extraction structures on 3 datasets using trial-wise *K*-fold validation. Statistical significance compared to the proposed DSE-TAM model is indicated by asterisks (* indicates *P* < 0.05, ** indicates *P* < 0.01, and *** indicates *P* < 0.001). Boldface indicates the best-performing result in each column.

Method	DEAP (A)/%	DEAP (V)/%	DREAMER (A)/%	DREAMER (V)/%	CEED (H/N/S)/%
STA-only	75.84 ± 4.59**	74.62 ± 4.15**	74.29 ± 5.89**	75.66 ± 8.05*	61.00 ± 12.01***
STM-only	68.20 ± 9.09***	59.53 ± 8.56***	63.28 ± 12.24***	63.63 ± 12.23***	44.43 ± 3.40***
STA + FFN	76.00 ± 4.92	74.00 ± 4.04**	78.84 ± 5.22	75.79 ± 5.31*	64.18 ± 7.57***
STM + FFN	73.26 ± 6.83**	70.89 ± 6.73***	75.56 ± 8.59*	74.44 ± 7.23**	56.03 ± 10.22***
STA + STM + FFN	75.52 ± 6.04*	72.72 ± 4.73***	77.61 ± 6.43	77.86 ± 5.89*	57.39 ± 11.40***
DSE-TAM (ours)	**79.75 ± 3.61**	**78.27 ± 5.01**	**80.55 ± 6.68**	**83.91 ± 7.07**	**73.75 ± 10.04**

DSE-TAM, Dynamic Spatial Embedding and Temporal Attention Mamba; A, arousal; V, valence; H/N/S, happy/neutral/sad; STM, Shallow Temporal Mamba; FFN, Feed-Forward Network

Our analysis reveals the specific limitations of nonhybrid models. STM-only models, constrained by sequential forward state transitions, struggle to enable the [CLS] token to effectively aggregate global information. In contrast, STA-only models leverage self-attention to capture this global context but are less adept at modeling local dependencies. The TAM architecture overcomes these issues by effectively integrating both strategies, a successful hybrid approach supported by findings in models like Jamba [38].

Furthermore, we examined the contribution of the FFN by creating variants that integrate it with our other temporal modules. The results indicate that while an FFN can enhance a simpler architecture like the STM-only model, it offers no significant performance gain to the already effective TAM architecture. This suggests that the FFN becomes redundant when temporal dynamics are comprehensively captured. Thus, the TAM design simplifies the architecture while simultaneously enhancing temporal feature extraction, leading to its superior performance.

### Interpretability and visualization

To validate the efficacy of the proposed DSE-TAM structure in EEG-based emotion recognition, we assessed its interpretability through functional connectivity analysis (Fig. 5) and spatio-temporal dynamics (Fig. 6).

**Fig. 5.**
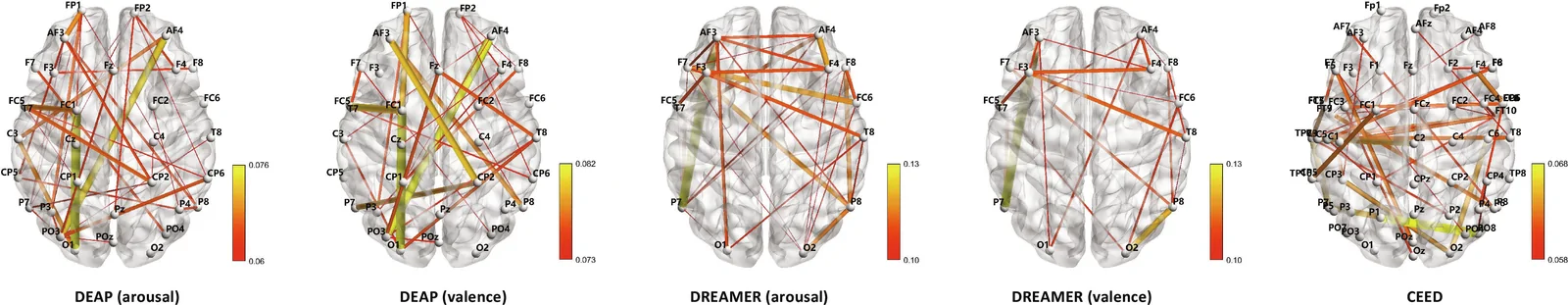
Connections in the learned adjacency matrix. The more yellow and the thicker the line, the higher the edge weight, indicating stronger connections between brain regions.

**Fig. 6.**
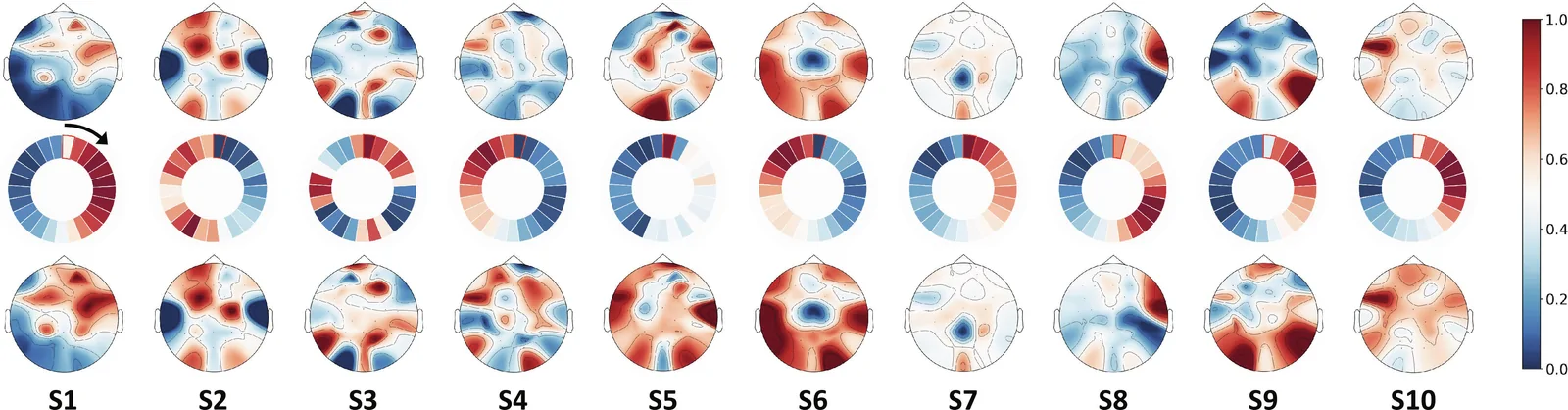
Topographic maps of feature weights are constructed using the first 10 subjects from the DEAP dataset. The first row presents the average topographical maps of electroencephalogram (EEG) signals after processing through brain network filters. The second row illustrates the temporal dynamics by showing the average token weights at different time points, arranged in a clockwise sequence. The third row integrates these token weights with the EEG signals to generate weighted topographical maps for each time point. a.u., arbitrary unit.

Fig. 5 visualizes the learned EEG channel adjacency matrices from the DSE module, highlighting salient inter-regional interactions. The DSE module effectively captures distinct, neuroscientifically plausible patterns for each dataset. In the DEAP dataset, connections are predominantly observed between the frontal and parietal cortices; DREAMER analysis reveals prominent links among frontal, temporal, and occipital lobes; and the CEED dataset exhibits a balanced interplay with numerous contralateral connections [39,40]. Crucially, robust frontal–posterior connections are consistently observed across all 3 datasets, underscoring a common connectivity pattern associated with visually evoked emotional activity [41,42]. These findings confirm the DSE module’s capacity to embed diverse and meaningful spatial patterns.

Fig. 6 illustrates the spatiotemporal dynamics learned by the Temporal Feature Extractor. The top row presents the baseline spatial topography, where consistent activation in the frontal and occipital lobes confirms their expected roles in emotion and visual processing [39,41]. The middle row displays the learned token weights over time, demonstrating that emotion elicitation is a temporally dynamic process with substantial intersubject variability [43]. Despite these variations, the [CLS] token, highlighted with a red frame, effectively aggregates information from the most salient temporal segments, serving as a stable anchor for capturing the signal’s spatiotemporal characteristics [44].

The bottom row of Fig. 6 shows the resulting weighted topographical maps, obtained by integrating the token weights with the corresponding EEG signals. Compared to the baseline maps, the weighted versions exhibit a marked enhancement of activation in key regions. Specifically, the model amplifies signals in the prefrontal cortex, associated with emotional regulation [45]; the lateral temporal regions, involved in processing emotional cues; and the parietal lobe, which contributes to emotion classification [39]. This demonstrates that the model effectively emphasizes features that are spatially and temporally aligned with key neural substrates of emotional processing. By dynamically weighting topographical representations, the proposed TAM enables accurate and focused emotion decoding based on salient neural patterns.

### Computational efficiency analysis

To further evaluate the practical applicability of the proposed framework, we compared DSE-TAM with representative baseline methods in terms of classification accuracy, multiply–accumulate operations (MACs), and parameter count. As shown in Fig. 7, DSE-TAM shows a favorable efficiency–performance trade-off across the DEAP, DREAMER, and CEED datasets. In the CEED subplot, the *x*-axis is displayed on a logarithmic scale because miMamba has substantially larger MACs than the other methods, which allows the remaining models to be compared more clearly.

**Fig. 7.**
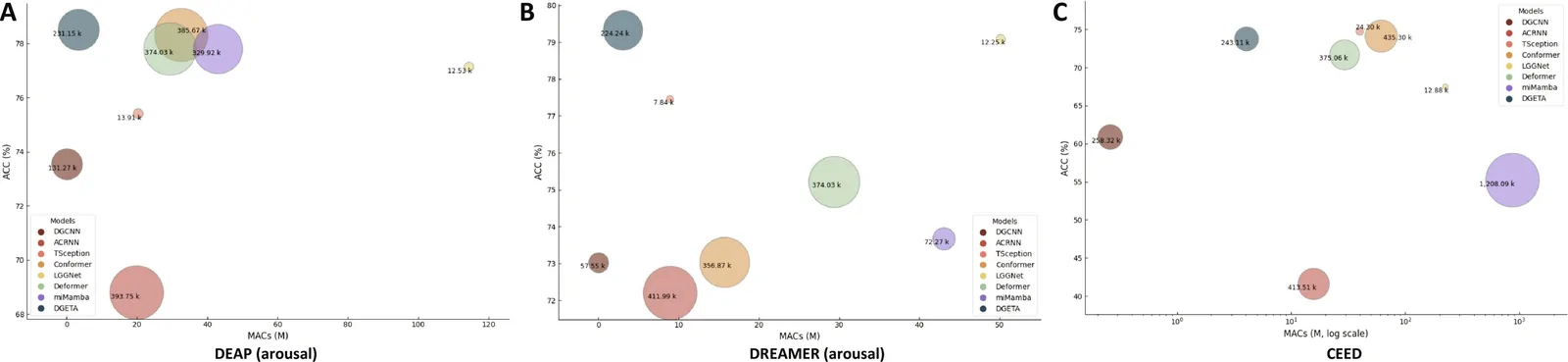
Efficiency comparison of Dynamic Spatial Embedding and Temporal Attention Mamba (DSE-TAM) and baseline methods on (A) DEAP, (B) DREAMER, and (C) CEED. The *y*-axis represents accuracy, the *x*-axis represents multiply–accumulate operations (MACs, in millions), and the size of each bubble corresponds to the number of parameters (in thousands).

Overall, models with heavier temporal modeling structures, such as Conformer and Deformer, tend to require higher computational costs, whereas lightweight models may reduce parameter size but do not always maintain competitive accuracy. Although lightweight models such as TSception and LGGNet may contain fewer parameters, their overall efficiency–performance trade-offs are not consistently superior. In contrast, DSE-TAM remains in a favorable region across different datasets, indicating that the proposed graph-guided spatial embedding and TAM-based temporal modeling can improve recognition performance while keeping the computational burden relatively low.

The comparison with miMamba is also informative, as both methods incorporate Mamba-based temporal modeling. However, miMamba shows a less favorable efficiency–performance trade-off, particularly on the CEED dataset with 63 EEG channels. Its markedly increased MACs suggest that its computational cost is more sensitive to high-channel EEG inputs, while its decoding performance on CEED remains limited. By contrast, DSE-TAM maintains competitive accuracy with relatively low MACs and a moderate parameter scale. This indicates that the proposed framework is more suitable for multichannel EEG emotion recognition scenarios where both accuracy and computational efficiency are important.

### Limitations and future work

Despite the promising overall performance of our proposed framework, several limitations warrant further investigation. First, the current implementation does not explicitly partition EEG channels based on spatial regions. This may limit its ability to fully capture the inherent local–global graph structure of EEG signals, thereby compromising the model’s capacity to represent intricate spatial dependencies [33,46]. Second, the Mamba module currently employs unidirectional state transitions, potentially restricting its ability to capture bidirectional temporal dependencies. Future work will explore extending Mamba to a bidirectional architecture to enhance the modeling of neural oscillations’ temporal dynamics and facilitate more comprehensive feature extraction [47]. Third, the classification accuracy for positive emotions, particularly on the CEED dataset, remains comparatively lower, likely stemming from the subtler neural correlates associated with positive emotional states. Addressing this issue may require more refined feature extraction techniques. Finally, the assumption that all samples within a trial share the same emotional label neglects potential within-trial emotion variations. To address this, we propose exploring a multi-instance learning approach, where trials are treated as bags of samples, to capture dynamic emotional transitions with greater fidelity [48].

## Conclusion

This paper introduces DSE-TAM, a novel and effective end-to-end model for EEG-based emotion recognition designed to address critical limitations in existing spatial and temporal modeling techniques. By leveraging GCNs to capture dynamic spatial relationships within EEG data and incorporating SSMs enhanced with self-attention mechanisms for superior temporal dynamics extraction, DSE-TAM effectively models both local and global dependencies inherent in EEG signals. Experimental evaluation on the DEAP, DREAMER, and CEED datasets demonstrates the superior performance of DSE-TAM. These advancements hold substantial potential for future applications in affective brain–computer interfaces, personalized healthcare, and other related domains where accurate and reliable emotion recognition is paramount. Future work will focus on exploring the framework’s generalizability across diverse populations and investigating its applicability in real-time emotion detection scenarios.

## Data Availability

The DREAMER dataset is accessible via https://zenodo.org/records/546113. The CEED dataset, which was collected by the authors, is available upon request to the corresponding authors. Any additional inquiries regarding the data can be directed to the corresponding authors.

## Supplementary Materials

Supplementary 1 Graphical Abstract Fig. S1 Tables S1 to S3
