# Supplementary material for: Beyond Static Representations: Learning Electroencephalogram Spatiotemporal Dynamics for Enhanced Emotion Recognition
Source: Cyborg Bionic Syst. 2026 Oct 28;7:0701. doi: 10.34133/cbsystems.0701 (PMC13714321; doi:10.34133/cbsystems.0701)
Supplement: Supplementary file 1 [file cbsystems.0701.f1.pdf]

Table S1 shows the parameter sensitivity analysis conducted on the DEAP dataset. We evaluated three key hyperparameters: the number of graph convolutional layers (**g_layers**), the embedding dimension (**emb_dim**), and the number of Temporal Aggregation Module (TAM) layers (**t_layers**). The analysis results show that 4 graph layers and an embedding dimension of 48 achieved the best performance on both Arousal (79.75%) and Valence (78.27%) tasks. For the number of TAM layers, 2 layers performed best on the Arousal task (79.75%), while 4 layers brought only a marginal performance improvement on the Valence task (78.80% vs 78.27%). Therefore, we ultimately selected **g_layers=4**, **emb_dim=48**, and **t_layers=2** as the model's final configuration, as it achieves the best trade-off between model performance and computational efficiency.

Table S1: Parameter sensitivity analysis on the DEAP dataset.

**Datasets**	**Metric**	**g_layers**			**emb_dim**			**t_layers**		
		2	4	6	32	48	64	1	2	4
DEAP	Arousal	78.46	**79.75**	77.80	78.47	**79.75**	78.83	76.90	**79.75**	79.40
	Valence	76.72	**78.27**	76.52	75.96	**78.27**	75.16	74.62	78.27	**78.80**

Table S2 presents the detailed performance comparison (Accuracy and F1-score) using trial-wise K-Fold cross-validation. Our proposed DSE-TAM model is benchmarked against six baseline methods (DGCNN, ACRNN, TSception, Conformer, LGGNet, and Deformer) across the DEAP, DREAMER, and CEED datasets. The results demonstrate that DSE-TAM achieves state-of-the-art performance, significantly outperforming all baselines on both Arousal and Valence tasks for the DEAP and DREAMER datasets. Specifically, on DEAP, our model achieves 79.75% accuracy for Arousal and 78.27% for Valence, and on DREAMER, it reaches 80.55% for Arousal and 83.91% for Valence. On the categorical CEED dataset, TSception achieves the highest performance (74.72% Acc), with our DSE-TAM (73.75%) and Conformer (74.12%) delivering highly competitive results. The statistical analysis (indicated by asterisks) confirms that the superiority of our model on DEAP and DREAMER is statistically significant ($p < 0.05$ or better) compared to most baseline models.

Table S2: Model performance (Accuracy and F1-score) using Trial-wise K-Fold Cross-Validation. *indicate $p < 0.05$, **indicate $p < 0.01$, ***indicate $p < 0.001$.

**Dataset**	**Task**	**Metric**	**DGCNN**	**ACRNN**	**TSception**	**Conformer**	**LGGNet**	**Deformer**	**miMamba**	**DSE-TAM (Ours)**
DEAP	Arousal	Acc	73.54 ± 5.22 ***	68.79 ± 12.32 ***	75.41 ± 6.01 **	78.32 ± 4.98	77.14 ± 6.04 *	77.80 ± 4.86 *	74.78 ± 7.38 ***	**79.75 ± 3.61**
		F1	76.17 ± 7.58	65.83 ± 23.05 ***	77.10 ± 12.00	77.90 ± 13.02	76.94 ± 13.76 *	77.45 ± 14.11	75.27 ± 12.31**	**80.10 ± 10.68**
	Valence	Acc	74.34 ± 6.00 **	65.84 ± 12.98 ***	74.85 ± 4.34 **	75.59 ± 3.10 **	73.84 ± 4.41 ***	75.71 ± 5.14 *	70.75 ± 4.73***	**78.27 ± 5.01**
		F1	74.50 ± 14.27*	66.33 ± 18.86 ***	76.47 ± 8.51 **	79.57 ± 5.09	75.40 ± 7.32 ***	77.56 ± 6.16 **	72.84 ± 8.91**	**80.62 ± 6.06**
DREAMER	Arousal	Acc	73.02 ± 6.29 ***	72.20 ± 7.33 ***	77.45 ± 7.36 *	73.03 ± 9.21 **	79.08 ± 6.21	75.21 ± 7.21 **	73.67 ± 6.02 ***	**80.55 ± 6.68**
		F1	62.46 ± 23.59 **	61.68 ± 20.81 **	74.16 ± 12.10	66.29 ± 15.22 **	72.66 ± 23.04	68.03 ± 15.09 **	68.44 ± 12.14 **	**75.92 ± 10.75**
	Valence	Acc	71.32 ± 9.54 ***	75.58 ± 6.56 ***	77.79 ± 9.28 ***	76.33 ± 7.01 ***	80.76 ± 7.43 *	76.18 ± 6.42 ***	75.19 ± 5.72 ***	**83.91 ± 7.07**
		F1	56.72 ± 19.15 **	56.08 ± 20.79 **	71.14 ± 15.62	68.48 ± 10.27 *	72.83 ± 10.23	66.32 ± 13.23 *	69.15 ± 8.35 *	**77.30 ± 20.97**
CEED	H/N/S	Acc	60.87 ± 10.48 ***	41.58 ± 14.72 ***	**74.72 ± 11.40**	74.12 ± 8.92	67.48 ± 9.11 ***	71.66 ± 10.33 *	55.21 ± 5.08 ***	73.75 ± 10.04
		F1	59.68 ± 11.62 ***	36.40 ± 16.16 ***	**74.19 ± 11.90**	73.60 ± 9.12	65.72 ± 10.12 ***	70.50 ± 11.36	48.86 ± 12.91 ***	71.50 ± 11.29

Table S3 summarizes the core contributions and limitations of the baseline models, which can be grouped into several architectural philosophies. A key finding is that many models have an imbalanced design. For instance, GNN-based models (DGCNN, LGGNet) excel at spatial modeling but are less adept at handling the complex, long-range temporal dependencies. Conversely, models in the CNN+Attention paradigm (Conformer, Deformer) are limited by their use of standard CNNs

for spatial extraction; their fixed, local receptive fields are inherently less effective at capturing the brain's dynamic, non-local functional connectivity. Other models show high specificity: TSception's multi-scale convolutions perform well on dense data but are less effective when adaptive, global connectivity is required. Finally, pure State-Space Models (miMamba) are constrained by their sequential mechanism, which, while strong temporally, is ineffective at modeling the non-local spatial relationships critical for EEG.

Table S3: Summary of Contributions and Limitations of Baseline Models.

**Algorithm**	**Contribution**	**Limitation**
DGCNN (Song. et. al, 2018)	Utilizes Graph Neural Networks (GNNs) with Differential Entropy (DE) features to model spatial relationships.	Less adept at handling the complex, long-range temporal dependencies in EEG sequences.
ACRNN (Tao. et. al, 2020)	Employs a hybrid structure (Attention + LSTM/RNN) to capture temporal dynamics.	Lacks a dedicated spatial module, making it less effective at capturing the brain's dynamic functional connectivity.
TSception (Ding. et. al, 2022)	Proposes multi-scale convolutions to extract rich temporal and spatial patterns at different scales.	The fixed-scale approach shows weaker generalization on sparse datasets and struggles to adaptively model global connectivity.
Conformer (Song. et. al, 2022)	Adapts the Conformer architecture (CNN + Self-Attention) to model both local and global temporal dependencies.	Uses standard CNNs for spatial extraction, which have fixed, local receptive fields and are less effective at capturing dynamic, non-local functional connectivity.
LGGNet (Ding. et. al, 2024)	A lightweight model that explicitly models both local and global spatial relationships via a Local-Global-Graph (LGG) module.	Primarily specializes in spatial modeling; its temporal components may be less effective for complex long-range dependencies.
Deformer (Ding. et. al, 2025)	Integrates a dense convolutional network with a Transformer to capture multi-scale features.	Similar to Conformer, its spatial modeling relies on standard CNNs, which are less effective than adaptive graph methods for dynamic connectivity.
miMamba (Zhuo. et. al, 2025)	Leverages State Space Models (SSMs) for efficient long-range temporal modeling, combined with Fast Fourier Transform(FFT).	Relies on a purely sequential architecture, making it ineffective at modeling non-local spatial connectivity and capturing comprehensive global representations.

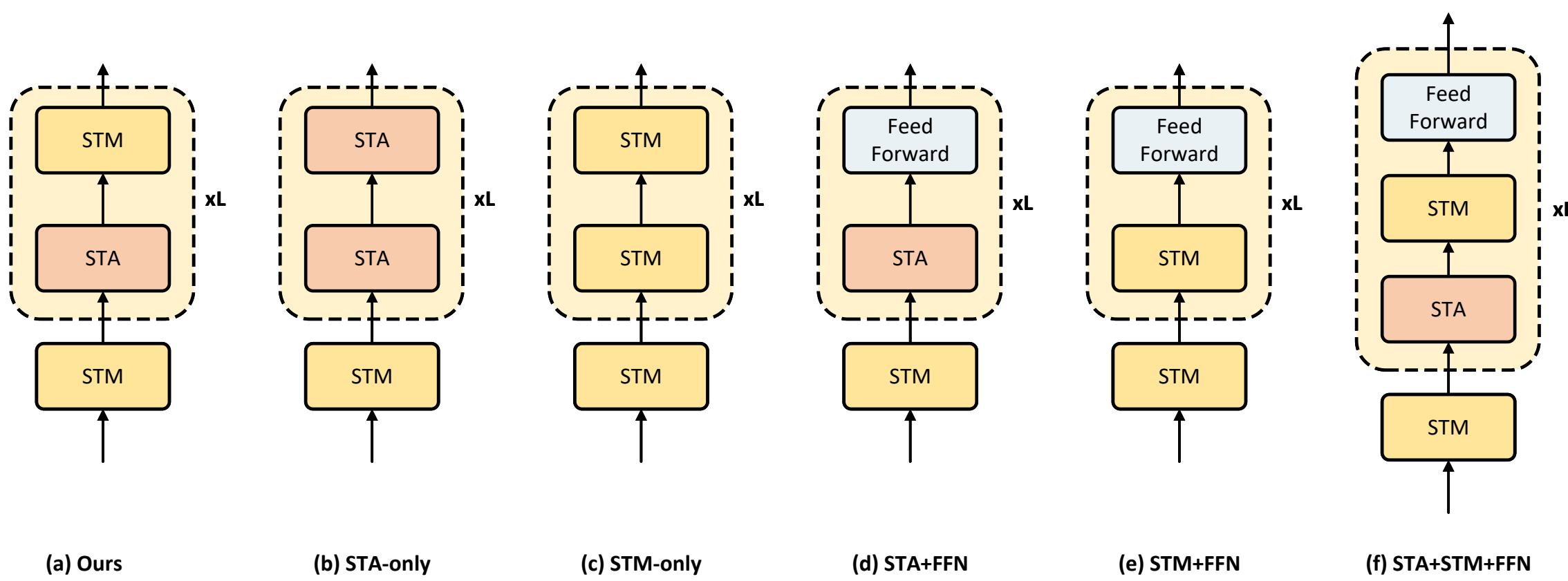

Figure S1: Architectures of different temporal feature extraction methods. The figure illustrates six different structures for modeling EEG temporal dynamics: (a) our proposed method (TAM), (b) STA-only model, (c) STM-only model, (d) STA+FFN model, (e) STM+FFN model, and (f) STA+STM+FFN model. Each method integrates different combinations of Selective Temporal Aggregation (STA), State Transition Model (STM), and Feedforward layers to capture local and global dependencies in EEG signals.
